# Relation between child maltreatment and human capital: results from a population-based birth cohort

**DOI:** 10.1590/0102-311XEN173623

**Published:** 2024-08-26

**Authors:** Roberta Hirschmann, Cauane Blumenberg, Pedro San Martin Soares, Ana Maria Baptista Menezes, Fernando César Wehrmeister, Helen Gonçalves

**Affiliations:** 1 Universidade Federal de Pelotas, Pelotas, Brasil.; 2 Causale Consultoria, Pelotas, Brasil.

**Keywords:** Child Abuse, Intelligence, Educational Status, Birth Cohort, Longitudinal Studies, Maus-Tratos Infantis, Inteligência, Escolaridade, Coorte de Nascimento, Estudos Longitudinais, Maltrato a los Niños, Inteligencia, Escolaridad, Cohorte de Nacimiento, Estudios Longitudinales

## Abstract

This study aimed to investigate the association between child maltreatment and human capital, measured by intelligence quotient (IQ) at age 18 years and schooling at age 22 years in 3,736 members from a population-based birth cohort in Southern Brazil. A multiple linear regression was used to assess the association between child maltreatment and human capital measurements. Physical and emotional abuse and physical neglect occurring up to 15 years of age were considered child maltreatment. Physical neglect was associated with lower IQ scores in women (β = -4.40; 95%CI: -6.82; -1.99) and men (β = -2.58; 95%CI: -5.17; -0.01) and lower schooling for all sexes: women (β = -1.19; 95%CI: -1.64; -0.74) and men (β = -0.82; 95%CI: -1.34; -0.30). Moreover, men who had experienced one type of child maltreatment and women who had experienced two or more types had lower years of schooling at 22 years (β = -0.41; 95%CI: -0.73; -0.89 and β = -0.57; 95%CI: -0.91; -0.22, respectively) than those who suffered no kind of maltreatment. Efforts to improve future educational and cognitive outcomes must include early prevention and intervention strategies for child maltreatment.

## Resumo

O objetivo deste estudo foi investigar a associação entre maus-tratos infantis e capital humano, medido por meio do quociente de inteligência (QI) aos 18 anos e escolaridade aos 22 anos, em 3.736 membros de uma coorte de nascimentos de base populacional no Sul do Brasil. A regressão linear múltipla foi utilizada para avaliar a associação entre maus-tratos infantis e medidas de capital humano. Foram considerados maus-tratos físicos, emocionais e a negligência física ocorrida até os 15 anos de idade. A negligência física associou-se a menores escores de QI entre mulheres (β = -4,40; IC95%: -6,82; -1,99) e homens (β = -2,58; IC95%: -5,17; -0,01), assim como menor escolaridade em mulheres (β = -1,19; IC95%: -1,64; -0,74 e homens (β = -0,82; IC95%: -1,34; -0,30). Além disso, homens que sofreram algum tipo de mau-trato na infância e mulheres que sofreram dois ou mais tipos apresentaram menor escolaridade aos 22 anos de idade (β = -0,41; IC95%: -0,73; -0,89 e β = -0,57; IC95%: -0,91; -0,22, respectivamente), em comparação com aqueles que não sofreram maus-tratos. Os esforços para aprimorar os futuros resultados educacionais e cognitivos devem incorporar estratégias de prevenção e intervenção precoce contra o mau-trato infantil.

## Resumen

El objetivo de este estudio fue investigar la asociación entre el maltrato infantil y el capital humano, medido utilizando el cociente de inteligencia (CI) a los 18 años y escolaridad a los 22 años, en 3.736 miembros de una cohorte de nacimientos de base poblacional en el Sur de Brasil. Se utilizó una regresión lineal múltiple para evaluar la asociación entre el maltrato infantil y las medidas de capital humano. Se consideraron el maltrato físico y emocional y la negligencia física que ocurrieron hasta los 15 años de edad. La negligencia física se asoció con puntuaciones de CI más bajas entre mujeres (β = -4,40; IC95%: -6,82; -1,99) y hombres (β = -2,58; IC95%: -5,17; -0,01), así como con una menor escolaridad en las mujeres (β = -1,19; IC95%: -1,64; -0,74 y hombres (β = -0,82; IC95%: -1,34; -0,30). Además, los hombres que sufrieron algún tipo de maltrato en la infancia y las mujeres que sufrieron dos o más tipos de maltrato tenían menor escolaridad a los 22 años (β = -0,41; IC95%: -0,73; -0,89 y β = -0,57; IC95%: -0,91; -0,22, respectivamente), en comparación con aquellos que no sufrieron maltratos. Los esfuerzos para mejorar los resultados educativos y cognitivos futuros deben incorporar estrategias de prevención e intervención temprana contra el maltrato infantil.

## Introduction

Human capital consists of a set of resources associated with knowledge and skills individuals acquire during their lives ^1^ ^,^ ^2^. Studies have used several instruments to evaluate this broad and multidimensional concept, including questionnaires and tests to assess the intelligence quotient (IQ), education, and income.

Increased human capital can be obtained with activities that improve health and education, reflecting good quality of work, increasing productivity, and, consequently, income, thus resulting in better living conditions for individuals, their families, and the well-being of society ^2^. However, situations experienced during the beginning of life, such as stressful events, are linked to future achievements and can negatively influence human capital gains ^3^ ^,^ ^4^. Child maltreatment is one of these events. It is defined as all forms of physical, emotional, sexual abuse and neglect that happen together or individually before 18 years of age and that harm the children’s health, survival, development, or dignity in a relationship of responsibility, trust, or power ^5^.

Children who have been abused may face difficulties socializing, concentrating, and acquiring new knowledge and skills ^6^ ^,^ ^7^ ^,^ ^8^, which compromises their human capital ^9^. Findings show that child maltreatment is associated with school absenteeism ^10^ ^,^ ^11^, lower IQ scores ^8^ ^,^ ^12^ ^,^ ^13^, reduced income, and education in adulthood ^12^. Furthermore, some evidence describes a dose-response relationship between a larger number of child maltreatment instances and increased social and health problems ^14^ ^,^ ^15^.

Most studies on this subject relied on small or convenience samples, and few have evaluated the relationship between child maltreatment and human capital according to sex ^16^. A recent meta-analysis concluded that the odds of absenteeism was higher in boys who experienced physical abuse and in girls who suffered sexual abuse in childhood, when compared to those who suffered no such child maltreatments ^16^, evincing that girls and boys respond differently to the stress resulting from child maltreatment ^9^ ^,^ ^16^.

Therefore, assessing the relationship between child maltreatment and outcomes related to human capital, especially stratified according to sex, remains important due to its high social, emotional, and financial cost for individuals, their families, and society. This study evaluated the association between child maltreatment (physical and emotional abuse and physical neglect) and human capital (IQ and schooling) in a population-based birth cohort in Brazil.

## Methods

### Sample

Data from the 1993 Pelotas (Brazil) birth cohort study, a longitudinal study that included all live births, from January 1st to December 31st, 1993, who lived in the urban area of the municipality of Pelotas (Rio Grande do Sul State), were used. Pelotas is a municipality in Southern Brazil that has about 344,000 inhabitants ^17^. Out of the 5,265 live births in the municipality in 1993, 5,249 mother-infant dyads agreed to participate in the study and were included in the sample. Mothers were interviewed shortly after delivery, and information on demographic, socioeconomic, and health-related variables was collected ^18^. All cohort members were followed-up when they reached a mean age of 11, 15, 18, and 22 years ^19^. More detailed information about the study methodology can be accessed in other publications ^18^ ^,^ ^19^ ^,^ ^20^.

Of the 5,249 included in the perinatal follow-up, 4,349 were followed up at age 15 years (85.7%); 4,106, at age 18 years (81.4%); and 3,810 at age 22 years (76.3%). The analytical sample of this study is composed of 3,736 (followed up at age 18 years) and 3,413 (followed up at age 22 years) individuals who had complete information regarding exposure and outcomes of interest.

### Human capital

Human capital was the outcome of interest of this study, measured by individuals’ IQ at age 18 years and schooling at age 22 years. The reduced version of the third version of the *Wechsler Adult Intelligence Scale* (WAIS-III) was used to measure IQ. It included four subtests: arithmetic, codes, similarities, and completing pictures ^21^. The raw scores of each subtest were weighted according to the Brazilian standard ^22^. The WAIS-III was administered individually in a private room by trained psychologists following the standard procedure. In turn, schooling was measured in complete years of study, which was self-reported by participants at age 22 years during follow-up.

### Child maltreatment

Exposure to child maltreatment up to age 15 years was collected at the 15-years-of-age follow-up by a questionnaire developed in REDCap (https://redcapbrasil.com.br/) by a computer-aided self-interview ^23^. Following previous research with this questionnaire ^24^, exposure to different child maltreatments was considered if an affirmative answer was provided to the following questions:

Physical abuse: “Has an adult in your family or someone who was taking care of you ever hit you in a way that left you bruised?”.

Emotional abuse: “Have you ever thought or felt that your father or mother didn’t want you to be born?” and/or “Have you ever thought or felt that someone in your family hates you?”. These occurrences were described as emotional “abuse” rather than “neglect” because the items seem to reflect acts of abuse committed against the child rather than omission (neglect).

Physical neglect: “Has it ever happened that you didn’t have enough food at home, or you wore dirty or torn clothes because you didn’t have any other clothes?”.

Each form of child maltreatment was evaluated dichotomously (yes/no). The number of child maltreatment instances was calculated by adding up the number of affirmative answers to each of the maltreatments (0, 1, 2 or more).

### Potential confounding variables

The following confounding variables were considered in the adjusted analyses of the association between child maltreatment and human capital: skin color/ethnicity (white, black, brown, yellow, Indigenous), family income (number of Brazilian minimum wages), mother’s and father’s schooling (complete years of study). All potential confounders were measured during the perinatal study.

### Statistical analysis

Chi-squared tests were fitted to examine differences between the analytic and original birth cohort samples regarding the distribution of sex, family income, mother’s age and schooling, and father’s schooling (all of which were perinatally measured) (Supplementary Material - Table S1; https://cadernos.ensp.fiocruz.br/static//arquivo/suppl-e00173623_1506.pdf).

Crude and adjusted linear regressions were used to examine the association of child maltreatment with IQ and schooling. The distribution of residuals was graphically tested. They showed a symmetric distribution. The variance inflation factor was assessed in the adjusted analyses, and no evidence of collinearity was found.

Analyses were also performed considering a possible interaction between child maltreatment and sex. Overall, two measurements of child maltreatment (emotional abuse and maltreatment score) were significant at 10% significance level (p < 0.10).

The significance level for association analyses was set at 5%, and all analyses were performed using Stata, version 14.0 (https://www.stata.com).

### Ethical aspects

The 1993 Pelotas (Brazil) birth cohort study was approved by the Research Ethics Committee of the Faculty of Medicine of the Federal University of Pelotas. To participate in all follow-ups, participants had to agree and sign an informed consent form - when participants were aged under 18 years, their caregivers also had to agree to participate by signing a form. Follow-ups at ages 15, 18, and 22 years were approved under protocols 40600026, 11/05, and 1250366, respectively.

## Results

Table 1 characterizes the analytical samples at the 18- and 22-years-follow-ups. Participants in both follow-ups were mostly white women who had a family income of up to three minimum wages. Regarding parents’ characteristics at birth, about 27% of mothers were aged from 20 to 24 years and half of the fathers had from 5 to 8 years of schooling. The mean IQ at age 18 years and schooling at age 22 years totaled 96.9 points (standard deviation - SD: ± 12.3) and 9.9 years of study (SD: ± 2.3), respectively.

**Table 1:** Characteristics of the samples at the 18- and 22-years-of-age follow-ups. 1993 Pelotas (Brazil) birth cohort study.

Characteristics	18 years (n = 3,736)			22 years (n = 3,413)		
	n	%	IQ Mean (95%CI)	n	%	Schooling Mean (95%CI)
General			96.9 (96.5; 97.2)			9.9 (9.8; 9.9)
Sex						
Male	1,767	47.3	96.6 (96.0; 97.2)	1,558	45.7	9.5 (9.3; 9.6)
Female	1,969	52.7	97.1 (96.6; 97.6)	1,855	54.3	10.2 (10.1; 10.3)
Skin color/Ethnicity						
White	2,406	64.4	99.2 (98.7; 99.0)	2,175	63.8	10.2 (10.1; 10.3)
Black	546	14.6	91.6 (90.7; 92.3)	515	15.1	9.1 (8.9; 9.3)
Brown	648	17.4	93.0 (92.4; 93.4)	593	17.4	9.2 (9.0; 9.4)
Yellow	58	1.8	95.6 (92.3; 98.9)	61	1.9	9.4 (8.8; 10.1)
Indigenous	68	1.8	95.4 (92.4; 98.3)	68	1.9	9.5 (9.0; 10.0)
Family income (minimum wages)						
≤ 1	651	17.7	91.0 (90.2; 91.9)	582	17.4	8.6 (8.4; 8.8)
1.1-3	1,557	42.4	95.5 (94.9; 96.1)	1,400	42.8	9.5 (9.4; 9.6)
3.1-6	902	24.6	98.8 (98.1; 99.6)	852	25.4	10.4 (10.3; 10.6)
6.1-10	293	8.0	103.4 (102.0; 104.7)	232	6.9	11.1 (10.9; 11.3)
> 10	268	7.3	106.2 (104.7; 107.7)	286	8.5	11.5 (11.4; 11.6)
Maternal age (years)						
< 20	635	17.0	94.2 (93.3; 95.1)	586	17.2	9.3 (9.1; 9.5)
20-24	1,026	27.5	96.2 (95.5; 97.0)	944	27.6	9.7 (9.6; 9.9)
25-29	964	25.8	97.2 (96.4; 98.0)	877	25.7	9.9 (9.8; 10.1)
30-34	699	18.7	98.7 (97.8; 99.6)	638	18.7	10.2 (10.0; 10.4)
≥ 35	411	11.0	98.8 (97.5; 100.0)	368	10.8	10.2 (10.0; 10.5)
Maternal schooling (years)						
0-4	987	26.5	90.8 (90.1; 91.5)	921	26.4	8.6 (8.4; 8.7)
5-8	1,803	48.3	96.3 (95.3; 96.8)	1,614	47.4	9.8 (9.7; 10.0)
≥ 9	940	25.2	104.4 (103.7; 105.1)	894	26.2	11.3 (11.2; 11.3)
Paternal schooling (years)						
0-4	869	24.9	92.3 (91.54; 92.1)	773	24.3	8.8 (8.7; 9.0)
5-8	1,734	49.8	96.1 (95.61; 96.7)	1,580	49.6	9.8 (9.7; 9.9)
≥ 9	883	25.3	104.1 (103.3; 104.9)	832	26.1	11.2 (11.0; 11.3)

95%CI: 95% confidence interval; IQ: intelligence quotient.

**Figure 1:**
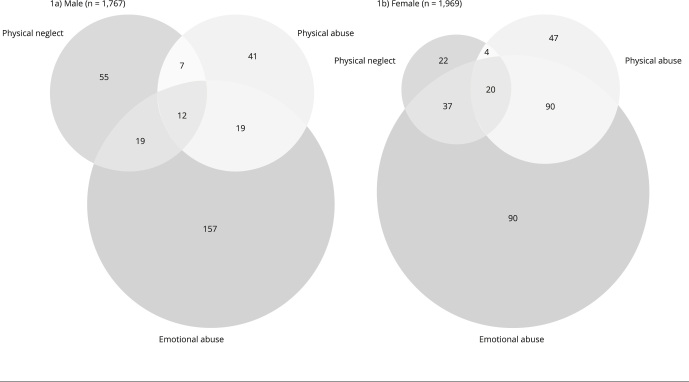
Venn diagram of child maltreatment occurring before of 15 years of age by sex. 1993 Pelotas (Brazil) birth cohort study (n = 3,736).

Figure 1 shows the distribution of child maltreatment. Physical and emotional abuse occurred more prevalently among women than in men. In general, emotional abuse was the most prevalent form of maltreatment, reported by around 27% of women and 13% of men.

Table 2 shows the associations between child maltreatment and IQ at age 18 years. In the adjusted analysis, women who experienced physical neglect before age 15 years averaged 4.4 IQ points lower than those who suffered no physical neglect (β = -4.40; 95% confidence interval - 95%CI: -6.82; -1.99). Men who experienced the same type of child maltreatment showed an average reduction of 2 .6 IQ points than those who suffered no physical neglect (β = -2.58; 95%CI: -5.17; -0.01).

**Table 2:** Association between child maltreatment instances before age 15 years and intelligence quotient (IQ) scores at age 18 years by sex. 1993 Pelotas (Brazil) birth cohort (n = 3,736).

	IQ			
	Men (n = 1,767)		Women (n = 1,969)	
	Crude	Adjusted *	Crude	Adjusted *
	β (95%CI)	β (95%CI)	β (95%CI)	β (95%CI)
Physical abuse	p = 0.288	p = 0.131	p = 0.672	p = 0.664
No	Reference	Reference	Reference	Reference
Yes	1.43 (-1.21; 4.09)	1.89 (-0.56; 4.35)	-0.40 (-2.27; 1.46)	0.38 (-1.34; 2.10)
Physical neglect	p < 0.001	p = 0.050	p < 0.001	p < 0.001
No	Reference	Reference	Reference	Reference
Yes	-7.22 (-9.94; -4.50)	-2.58 (-5.17; -0.01)	-6.59 (-9.13; -4.05)	-4.40 (-6.82; -1.99)
Emotional abuse	p = 0.808	p = 0.398	p = 0.006	p = 0.095
No	Reference	Reference	Reference	Reference
Yes	0.22 (-1.60; 2.05)	0.72 (-0.95; 2.40)	-1.62 (-2.78; -0.47)	-0.90 (-1.97; 0.15)
Child maltreatment score	p = 0.261	p = 0.957	p = 0.002 **	p = 0.127 **
0	Reference	Reference	Reference	Reference
1	-1.36 (-3.11; 0.39)	0.23 (-1.38; 1.86)	-1.08 (-2.31; 0.15)	-0.62 (-1.75; 0.51)
2 or more	-1.12 (-4.12; 1.88)	0.13 (-2.64; 2.91)	-3.30 (-5.25; -1.35)	-1.73 (-3.55; 0.81)

95%CI: 95% confidence interval.

* Linear regression adjusted for family income (minimum wages), skin color/ethnicity, maternal schooling, and paternal schooling;

** p-value of the linear trend test.

In both sexes, those who suffered physical neglect averaged about one year less of schooling than those unexposed to this type of maltreatment (women β = - 1.19; 95%CI: -1.64; -0.74 and men β = -0.82; 95%CI: -1.34; -0.30). Lower schooling also occurred in women who suffered emotional abuse (β = -0.23; 95%CI: -0.43; -0.03). Moreover, men who experienced one type of child abuse and women who experienced two or more types had half a year less schooling than those who suffered no child abuse (β = -0.41; 95%CI: -0.73; -0.89 and β = -0.57; 95%CI: -0.91; -0.22, respectively) (Table 3).

**Table 3:** Association between child maltreatment instances before age 15 years and schooling at 22 years by sex. 1993 Pelotas (Brazil) birth cohort study (n = 3,413).

	Schooling			
	Men (n = 1.558)		Women (n = 1.855)	
	Crude	Adjusted *	Crude	Adjusted *
	β (95%CI)	β (95%CI)	β (95%CI)	β (95%CI)
Physical abuse	p = 0.704	p = 0.774	p = 0.143	p = 0.419
No	Reference	Reference	Reference	Reference
Yes	-0.10 (-0.64; 0.43)	0.07 (-0.41; 0.55)	-0.27 (-0.63; 0.09)	-0.13 (-0.46; 0.19)
Physical neglect	p < 0.001	p = 0.002	p < 0.001	p < 0.001
No	Reference	Reference	Reference	Reference
Yes	-1.89 (-2.44; -1.33)	-0.82 (-1.34; -0.30)	-1.67 (-2.14; -1.19)	-1.19 (-1.64; -0.74)
Emotional abuse	p = 0.021	p = 0.143	p < 0.001	p = 0.021
No	Reference	Reference	Reference	Reference
Yes	-0.43 (-0.80; -0.06)	-0.25 (-0.58; 0.08)	-0.45 (-0.67; -0.23)	-0.23 (-0.43; -0.03)
Child maltreatment score	p < 0.001	p = 0.037	p < 0.001 **	p = 0.002 **
0	Reference	Reference	Reference	Reference
1	-0.78 (-1.14; -0.43)	-0.41 (-0.73; -0.89)	-0.34 (-0.57; 0.11)	-0.16 (-0.37; 0.04)
2 or more	-0.65 (-1.25; -0.04)	-0.21 (-0.76; 0.33)	-0.93 (-1.30; -0.56)	-0.57 (-0.91; -0.22)

95%CI: 95% confidence interval.

* Linear regression adjusted for family income (minimum wages), skin color/ethnicity, maternal schooling, and paternal schooling;

** p-value of the linear trend test.

## Discussion

This study evaluated the association between child maltreatment and human capital in early adulthood. Our results showed differences between sexes. IQ was only negatively associated with physical neglect, and schooling was negatively associated with physical neglect and emotional abuse in women and only with physical neglect in men.

The literature has explored the association of child maltreatment with cognitive and educational outcomes less than other outcomes, such as mental health and substance abuse. However, some evidence shows that individuals who suffer abuse or neglect during their childhood had lower IQ scores and schooling ^8^ ^,^ ^12^ ^,^ ^13^ ^,^ ^25^ ^,^ ^26^ ^,^ ^27^, and that the negative effect of maltreatment can also be observed on offsprings’ educational outcomes ^28^. Although less evaluated in the literature, physical neglect can be as harmful as other types of child maltreatment. It can reduce cognitive outcomes due to the lack of an environment that encourages children to reach their full potential and attentive parents to provide the basic needs of their children ^10^ ^,^ ^27^ ^,^ ^28^ ^,^ ^29^. Studies suggest that repeated or long-lasting stress during sensitive developmental stages in childhood or adolescence promotes long-term adverse biological effects (particularly in limbic and prefrontal areas) that might persist until adulthood ^29^ ^,^ ^30^. These alterations usually follow cognitive deficits in attention, intelligence, and working memory ^30^ ^,^ ^31^ and may cause worse educational results ^8^ ^,^ ^9^.

This study associated the occurrence of physical neglect before age 15 years with lower schooling at age 18 years in women and men. Individuals of both sexes who endured physical neglect had lower schooling compared to those who experienced no such situations. Longitudinal studies have suggested a negative effect of the exposure to physical neglect early in life (before 15 years) on subsequent cognitive and educational impairments ^12^ ^,^ ^27^ ^,^ ^32^ ^,^ ^33^ ^,^ ^34^. This study found no studies in the literature that evaluated these associations according to sex.

The association between physical neglect and IQ may be explained by the differences in the perception of physical negligence according to the sex of the victim. According to Cutler & Nolen-Hoeksema ^35^, girls are more likely to blame themselves for stressful life events, including neglect and abandonment, causing greater vulnerability to emotional and cognitive problems in life. A biological explanation for the observed sex difference focuses on genetic predispositions influencing hormonal systems ^36^. For example, men have higher concentrations of testosterone, whereas women, of estrogens ^37^. Such differences may moderate how women and men respond to repeated neglect ^38^, with greater damage to women due to sociocultural issues, such as the social role of gender culturally advocated for both.

Results also showed an association between higher maltreatment scores (one type of abuse for men and two or more for women) and fewer years of schooling at age 22 years. Some studies have shown a negative effect of an increased number of adversities in childhood and the concomitant effect of exposure to child maltreatment ^34^ ^,^ ^39^ ^,^ ^40^ on lower schooling. However, no studies stratified this effect by sex. Hence, this study seeks to fill this gap in the literature.

Some strengths of this study deserve to be pointed out. First, it used longitudinal data, which offers well-known advantages over cross-sectional data, such as ensuring the timing between exposure and outcome and the long-term effect that child maltreatment might have on human capital. Second, this study analyzed a population-based birth cohort from a medium-sized municipality with characteristics that resembled that of other municipalities in Brazil. Thus, may be possible to extrapolate its findings to other medium- and small-sized Brazilian cities, filling an important gap in the current knowledge about the relationship between child maltreatment and human capital.

Moreover, data were collected in a standardized fashion across follow-ups by computer-aided self-interviews that aimed to reduce the probability of occurrence of the ‘social desirability’ bias during the investigation of sensitive subjects ^41^.

However, some limitations apply to this study. First, the noninclusion of sexual abuse as one of its exposures refers to the high degree of uncertainty in its estimates. It is important to mention that its low prevalence might be due to underreporting. However, all child maltreatment variables were assessed by a confidential self-administered questionnaire, minimizing the underreporting bias. Also, as the occurrence of abuse was retrospectively collected, it may lead to underreporting, reducing its effect measurement due to recall bias. However, these cases of violence (physical and emotional abuse and/or neglect) strongly impact the lives of individuals, reducing this bias.

This study also highlights that the lack of information on the frequency, period, severity, and duration of exposure to child abuse hinders accurately estimating its occurrence.

Maltreatment experienced during early life, especially neglect, affects intelligence and is related to reduced schooling later in life. Negligence is a less studied type of child abuse but it should receive more attention in new prospective studies on this subject due to its importance.

Child abuse has negative consequences and serious implications for victims, families, the social groups they inhabit, and institutions. Thus, efforts to improve future outcomes and enable individuals to reach their maximum potential in adulthood should include prevention strategies and early intervention of negative adverse events during childhood, such as the cases of abuse and neglect in this study.
