# Supplementary material for: Relation between child maltreatment and human capital: results from a population-based birth cohort
Source: Cad Saude Publica. 2024 Aug 26;40(7):e00173623. doi: 10.1590/0102-311XEN173623 (PMC11349277; doi:10.1590/0102-311XEN173623)
Supplement: Supplementary file 1 [file 1678-4464-csp-40-07-EN173623-s.pdf]

SUPPLEMENTARY MATERIAL

Table S1 Characteristics of the original sample and of the analyzed sample from the cohort.

Characteristics	Original sample (n = 5,249)	Sample study	
		18 years (n = 3,736)	22 years (n = 3,413)
	n (%)	n (%)	n (%)
Sex			
Male	2,575 (49.5)	1,767 (47.3)	1,558 (45.6)
Female	2,632 (50.6)	1,969 (52.7)	1,855 (54.4)
Family income (minimum wages)			
≤ 1	949 (18.6)	651 (17.7)	582 (17.4)
1.1-3	2,139 (42.0)	1,557 (42.4)	1,400 (41.7)
3.1-6	1,197 (23.5)	902 (24.6)	852 (25.4)
6.1-10	429 (8.4)	293 (8.0)	268 (8.0)
> 10	384 (7.5)	268 (7.3)	250 (7.5)
Maternal schooling (years)			
0-4	1,449 (27.9)	987 (26.5)	901 (26.4)
5-8	2,410 (46.3)	1,803 (48.3)	1,614 (47.4)
≥ 9	1,343 (25.8)	940 (25.2)	894 (26.2)
Paternal schooling (years)			
0-4	1,242 (25.5)	869 (24.9)	773 (24.3)
5-8	2,353 (48.5)	1,734 (49.8)	1,580 (49.6)
≥ 9	1,260 (26.0)	883 (25.3)	832 (26.1)
Maternal age (years)			
< 20	909 (17.5)	635 (17.0)	586 (17.2)
20-24	1,438 (27.6)	1,026 (27.5)	944 (27.6)
25-29	1,341 (25.8)	964 (25.8)	877 (25.7)
30-34	949 (18.2)	699 (18.7)	638 (18.7)
≥ 35	570 (11.0)	411 (11.0)	368 (10.8)
